# A Simple and Rapid Method for DNA Isolation from Xylophagous Insects

**DOI:** 10.3390/ijms11125056

**Published:** 2010-12-07

**Authors:** Nancy Calderón-Cortés, Mauricio Quesada, Horacio Cano-Camacho, Guadalupe Zavala-Páramo

**Affiliations:** 1 Center for Ecosystems Research (CIEco), National Autonomous University of Mexico (UNAM), Morelia, Mexico; E-Mail: mquesada@oikos.unam.mx (M.Q.); 2 Multidisciplinary Center of Biotechnology Studies (CMEB), Michoacan University of San Nicolas of Hidalgo (UMSNH), Morelia, Mexico; E-Mails: hcano1gz1@me.com (H.C.-C.); gzavpar@hotmail.com (G.Z.-P.)

**Keywords:** Cerambycidae, DNA isolation, PCR, Oncideres albomarginata chamela, phenolics, restriction digestion, xylophagous insects

## Abstract

Published methods to isolate DNA from insects are not always effective in xylophagous insects because they have high concentrations of phenolics and other secondary plant compounds in their digestive tracts. A simple, reliable and labor-effective cetyltrimethylammonium bromide-polyvinylpyrrolidone (CTAB-PVP) method for isolation of high quality DNA from xylophagous insects is described. This method was successfully applied to PCR and restriction analysis, indicating removal of common inhibitors. DNA isolated by the CTAB-PVP method could be used in most molecular analyses.

## 1. Introduction

Insect species that directly feed on wood play important functional roles in forest ecosystems, as they contribute to nutrient cycling [1–3]. Moreover, most species can be serious pests of forests and wood products, having economically important consequences for the forest and timber industries [4,5]. Due to their ecological and/or economical importance, research on systematics, phylogenetics, ecological genetics and molecular methods for detection and control of insect pests are needed [6,7]. For most molecular studies, the isolation of high quality DNA is an essential prerequisite. Nevertheless, the isolation of high quality DNA from xylophagous insects is usually cumbersome, because xylophagous insects tend to concentrate high amounts of plant phenolics and tannins in their digestive tracts [8,9]. Most of the published methods for insect DNA isolation are SDS/proteinase K based protocols [10–14] and commercially available kits [15,16]. These methods usually use adult specimens or specific tissues from thorax, head, wings or leg muscles to avoid contaminants. However, these methods are not useful for the isolation of high quality DNA due to the presence of phenolics and other plant contaminants in the digestive tract of xylophagous insects. Isolation of DNA is difficult from the tissue in the digestive tract [14,17], or when insects are too small to dissect them. Additionally, SDS based methods and commercially available kits tend to produce low DNA yields with short storage life from tissues rich in phenolics [18,19], which make them unsuitable for some molecular applications (e.g., Southern blot analysis, construction of genomic libraries, DNA fingerprinting, *etc*.).

Phenolics are recognized as the major contaminants in DNA preparations from plants [18,20,21]. Phenolics, as powerful oxidizing agents, can reduce the yield and purity of DNA by binding covalently with the extracted DNA, thereby inhibiting further enzymatic modifications of the DNA such as restriction endonuclease digestion and polymerase chain reaction (PCR) [18,22–25]. Higher concentrations of cetyltrimethylammonium bromide (CTAB) and the addition of antioxidants such as polyvinyl-pyrrolidone (PVP) and β-mercaptoethanol to the extraction buffer can help to remove phenolics in DNA preparations from plants [18,21,22,26,27]. However, PVP usually is not used in the methods reported for DNA isolation from insects [10–13,17,28].

We evaluated the effectiveness of the traditionally used CTAB method for isolation of DNA from the xylophagous insect, *Oncideres albomarginata chamela* (Coleoptera: Cerambycidae). In this study, we developed an inexpensive and rapid DNA isolation method by modifying several existing methods [18,21,22,26,27,29] for plant DNA isolation. We evaluated the quality of the DNA isolated using this modified method by restriction endonuclease digestion and PCR. The isolated DNA was suitable for these molecular applications. The CTAB-PVP method was also used for DNA isolation in three additional xylophagous beetles: *Ataxia alpha*, *Estoloides chamelae* and *Lissonotus flavocinctus* (Cerambycidae), confirming that this modified method can be applicable to other xylophagous insects.

## 2. Results and Discussion

**Figure 1.**
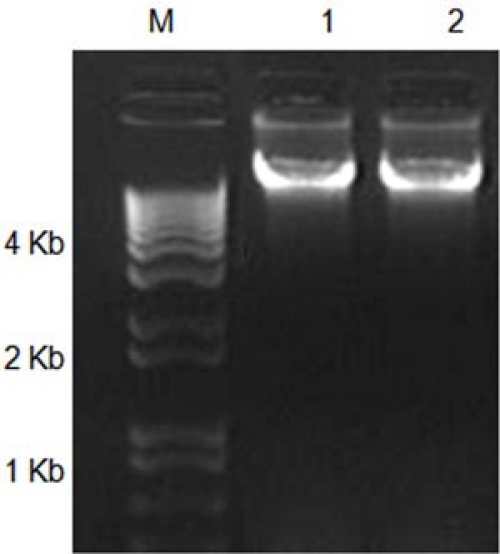
Agarose gel analysis of DNA prepared from *Oncideres albomarginata chamela* larvae with two DNA isolation methods. M, DNA size marker (1 Kb plus DNA ladder, Invitrogen, Carlsbad, CA, USA); Lane 1, genomic DNA isolated with the CTAB method; Lane 2, genomic DNA isolated with the CTAB-PVP method.

The type of contaminations arising in DNA isolated from biological material varies according to its origin (e.g., organism, tissue, life stage) [11,13,23]. Therefore, the type and condition of specimens and tissues are key factors in selecting a DNA isolation method. Tissues in the digestive tracts of xylophagous insects are rich in phenolics and tannins. These secondary compounds must be removed to obtain DNA free from contaminants. Phenolics and other secondary compounds cause damage to DNA and/or inhibit restriction endonucleases and Taq polymerases [18,23–25,27]. The widely used CTAB method occasionally fails to remove all phenolics from DNA preparations [18]. Antioxidants are commonly used to address problems related to phenolics; examples include β-mercaptoethanol, PVP, bovine serum albumin (BSA), among others [19,30]. PVP forms complex hydrogen bonds with phenolics and co-precipitates with cell debris upon cell lysis [18,21,31]. These PVP-phenolic complexes accumulate at the interface between the organic and aqueous phases and can be eliminated from DNA preparations. On the other hand, high concentrations of β-mercaptoethanol, helps to reduce the browning in DNA preparations produced by the oxidation of phenolics [22,27]. To test the effect of the inclusion of PVP and an increased concentration of β-mercaptoethanol in our DNA isolation method, we compared this method with the traditionally used CTAB method [29]. The results indicated similar yields (∼50 μg/100 mg fresh tissue) of high molecular weight DNA using both methods (Figure 1). Nevertheless, the A260/280 ratio for the CTAB method (1.21–1.32) and for the CTAB-PVP modified method (1.69–1.76) indicated a higher level of contamination in the DNA isolated by the traditional CTAB method.

**Figure 2.**
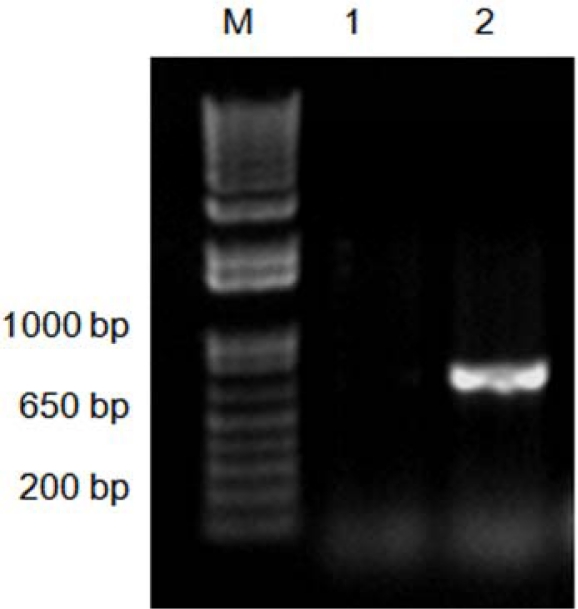
Amplification of a mitochondrial cytochrome oxidase I (COI) gene fragment using DNA (isolated from *Oncideres albomarginata chamela* larvae) that had been stored for three months. M, DNA size marker (1 Kb plus DNA ladder, Invitrogen, Carlsbad, CA, USA); Lane 1, genomic DNA isolated with the CTAB-method; Lane 2, genomic DNA isolated with the CTAB-PVP method.

**Figure 3.**
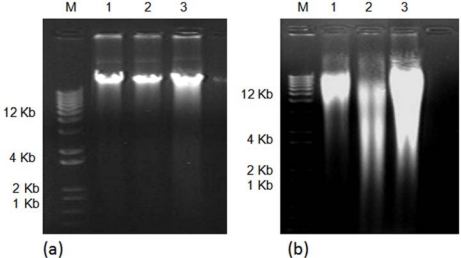
Analysis of *Oncideres albomarginata chamela-*DNA digested with different restriction enzymes and separated by 1% agarose gel electrophoresis. (**a**) Genomic DNA isolated with the CTAB-method, and (**b**) Genomic DNA isolated with the CTAB-PVP method. For both (a) and (b), Lane M, DNA size marker (1 Kb plus DNA ladder; Invitrogen, Carlsbad, CA, USA); Lane 1, restriction digestion with *Xba*I; Lane 2, restriction digestion with *Not*I; Lane 3, restriction digestion with *Eco*RI.

**Figure 4.**
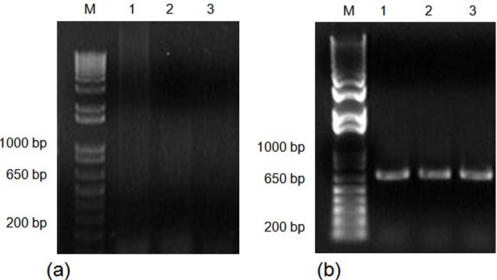
Amplification of a COI gene fragment using DNA isolated from larvae of three xylophagous beetles, after storage for three months. (**a**) Genomic DNA isolated with the CTAB method, and (**b**) Genomic DNA isolated with the CTAB-PVP method. For both (**a**) and (**b**), Lane M, DNA size marker (1 Kb plus DNA ladder; Invitrogen, Carlsbad, CA, USA); Lane 1, *Ataxia alpha*; Lane 2, *Estoloides chamelae;* Lane 3, *Lissonotus flavocinctus*.

**Figure 5.**
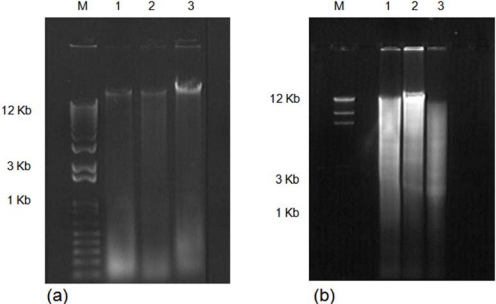
Analysis of restriction endonuclease digestion with *Eco*RI of the DNA of three xylophagous beetles, and separated by 1% agarose gel electrophoresis. (**a**) Genomic DNA isolated with the CTAB method, and (**b**) Genomic DNA isolated with the CTAB-PVP method. For both (**a**) and (**b**), Lane M, DNA size marker (1 Kb plus DNA ladder; Invitrogen, Carlsbad, CA, USA); Lane 1, *Ataxia alpha*; Lane 2, *Estoloides chamelae*; Lane 3, *Lissonotus flavocinctus.*

The isolated DNA using both methods was tested for PCR amplification. Amplifications of a mitochondrial cytochrome oxidase I (COI) gene fragment using fresh DNA obtained with both methods were successfully achieved. However, amplification of the COI gene fragment was observed only for the CTAB-PVP isolated-DNA after the DNA samples had been stored for three months (Figure 2). These results indicate that DNA isolated by the traditional CTAB method is not suitable for longer storage periods. Similar results have been previously reported [18,22]. DNA preparations containing contaminants have a shorter storage lifespan [18]. The most common contaminants are polysaccharides, RNA and phenolics [10–12,18–22,25,30,31]. Polysaccharides and phenolics usually produce highly viscous and brown-colored solutions, respectively [10,20,30]. Given that RNA contamination is normally removed by treatment with RNase [30], and the isolated DNA was not viscous, it is likely that phenolics are the contaminants present in the CTAB isolated-DNA. In addition, the inclusion of PVP and β-mercaptoethanol cleared the DNA solutions. This suggests that DNA isolated by the CTAB-PVP method had lower concentrations of phenolics compared with the traditionally used CTAB method. The purity and quality of the isolated DNA was also validated by digestion with different restriction endonucleases. The results showed a complete digestion of CTAB-PVP isolated-DNA (Figure 3a), while CTAB isolated-DNA showed only partial digestion (Figure 3a), indicating the presence of contaminants in this DNA preparation. The CTAB-PVP method demonstrated to be applicable to other xylophagous insects, since isolated DNA from three additional species of xylophagous beetles proved amenable for PCR amplification (Figure 4a) and restriction digestion (Figure 5), whereas DNA isolated with the CTAB-method was not suitable for PCR amplification (Figure 4a) and showed only partial digestion (Figure 5a).

## 3. Experimental Section

### 3.1. DNA Isolation

For DNA isolation, we used larvae at the last instar of the borer beetle *Oncideres albomarginata chamela*, because this life stage presents the highest concentration of phenolics and other plant contaminants [32]. DNA was isolated using CTAB [29] and a CTAB-PVP modified method. A mortar and pestle with the addition of liquid nitrogen were used for the grinding of fresh sample-tissue (100 mg) into fine powder. The ground tissue was transferred to a 1.5 mL tube and homogenized in 1 mL of prewarmed (60 °C) extraction buffer (20 mM ethylene diamide tetraacetic acid (EDTA) pH 8.0, 100 mM Tris-HCl pH 7.5, 1.4 M NaCl, 2% w/v CTAB, 4% w/v PVP-40). β-mercaptoethanol (2% v/v) was added to the extraction buffer just prior to use. Samples were incubated at 60 °C for 30 min with occasional mixing, and cooled to room temperature. Two microliters of RNase (1 mg/mL) were added to the solution and incubated at 37 °C for 15 min. One volume of chloroform:isoamyl alcohol (24:1) was added, and the sample was emulsified by gentle inversion and centrifuged for 15 min at 13,000 rpm. The top aqueous phase was transferred to a clean tube. A second chloroform:isoamyl extraction was performed when the aqueous phase was cloudy due to the presence of PVP. Two volumes of cold (−20 °C) 95% ethanol were added to the sample, mixed well and incubated at −20 °C until DNA strands were visible. DNA strands were recovered using a sterile Pasteur pipette and washed with 70% ethanol, centrifuged for 5 min at 13000 rpm, dried and finally eluted in sterile analytic grade H_2_O.

### 3.2. Comparison of the Efficacy for the DNA Isolation Methods

Electrophoresis was conducted using 1% TAE agarose gels. Gels were stained with ethidium bromide, and visualized under UV light. The quality of DNA isolated by the CTAB traditional method and the CTAB-PVP modified method was estimated by measuring the A260/280 absorbance ratio using a spectrophotometer (Perkin-Elmer Corp., Norwalk, CT, USA). A fragment of ∼650 base pairs of the COI gene (corresponding to the DNA universal barcoding region) was amplified from the freshly isolated DNA and DNA that had been stored for three months. The forward primer LCO (5′-GGTCAACAAATCATAAAGATATTGG-3′) and reverse primer HCO (5′-TAAACTTCAGGGTGACCAAAAAATCA-3′) were used for this purpose. PCR amplifications were carried out using a ramping-down of the annealing temperature (‘touchdown’; −2 °C per every five cycles) program. PCR amplification was started using the following settings: 94 °C/30 s (denaturing), 50 °C/30 s (base annealing temperature of first three cycles) and 72 °C/1 min (extension), and was continued until the base annealing temperature reached the final condition of 44 °C. Under the final conditions, the amplification was continued for 20 cycles. Additionally, the quality of the DNA isolated by both methods was evaluated by restriction analysis, for which 10 μg of DNA isolated by each method was incubated overnight with 10 U *Xba*I, *Not*I and *Eco*RI, and analyzed on 1% agarose gels.

### 3.3. Evaluation of the CTAB-PVP Modified Method in Other Xylophagous Species

To test if the modified method is applicable to other xylophagous insects, we isolated DNA from larvae of three additional cerambycid xylophagous beetles, *Ataxia alpha*, *Estoloides chamelae* and *Lissonotus flavocinctus* using the CTAB [29] and CTAB-PVP modified method as previously described. DNA isolated from each species was stored at −20 °C for three months. After the storage period, DNA was digested with *Eco*RI, and used as template for PCR amplification as previously described.

## 4. Conclusions

The modified CTAB-PVP method for DNA isolation seems to be suitable for PCR and restriction analyses. This method is rapid, simple and efficient for the isolation of DNA from xylophagous insects which possess high concentrations of plant compounds that can interfere with DNA extraction and analysis.

## References

[1] Amman GD, Mattson WJ (1976). The role of the mountain pine beetle in lodgepole pine ecosystems: impact on sucession. The Role of Arthropods in Forest Ecosystems.

[2] Schowalter TD (1981). Insect herbivore relationship to the state of the host plant: biotic regulation of the ecosystem nutrient cycling through ecological succession. Oikos.

[3] Feller IC (2002). The role of herbivory by wood-boring insects in mangrove ecosystems in Belize. Oikos.

[4] Allison JD, Borden JH, Seybold ST (2004). A review of the chemical ecology of the Cerambycidae (Coleoptera). Chemoecology.

[5] Knizek M, Beave R, Lieutier F, Day KR, Battisti A, Grégoire JC, Evans H (2004). Taxonomy and systematic of bark and Ambrosia beetles. Bark and Wood-Boring Insects in Living Trees in Europe, a Synthesis.

[6] Caterino MS, Cho S, Sperling FAH (2000). The current state of insect molecular systematics: a thriving tower of Babel. Annu. Rev. Entomol.

[7] Brockerhoff EG, Liebhold AM, Jactel H (2006). The ecology of forest insect invasions and advances in their management. Can. J. Forest Res.

[8] Strauss SY, Zangerl AR, Herrera CM, Pellmyr O (2002). Plant-insect interactions in terrestrial ecosystems. Plant-Animal Interactions: An Evolutionary Approach.

[9] Chown SL, Nicolson SW (2004). Insect Physiological Ecology.

[10] Henry JM, Raina AK, Ridgway RL (1990). Isolation of High Molecular-weight DNA from insects. Anal. Res.

[11] Aljanabi SM, Martinez I (1997). Universal and rapid salt-extraction of high quality genomic DNA for PCR-based techniques. Nucleic Acids Res.

[12] Reineke A, Karlovsky P, Zebitz PW (1998). Preparation and purification of DNA from insects for AFLP analysis. Insect Mol. Biol.

[13] Hill CA, Gutierrez JA (2003). A method for extraction and analysis of high quality genomic DNA from ixodid ticks. Med. Vet. Entomol.

[14] Juen A, Traugott M (2006). Amplification facilitators and multiplex PCR: tools to overcome PCR-inhibition in DNA-gut-content analysis of soil-living invertebrates. Soil Biol. Biochem.

[15] Stone GN, Challis RJ, Atkinson RJ, Csóka G, Hayward A, Melika G, Mutun S, Preuss S, Rokas A, Sadeghi E, Schönrogge K (2007). The phylogeographical clade trade: tracing the impact of human-mediated dispersal on the colonization of northern Europe by the oak gallwasp *Andricus kollari*. Mol. Ecol.

[16] Ball SL, Armstrong KF (2008). Rapid, one step DNA extraction for insect pest identification using barcodes. J. Econ. Entomol.

[17] Serrano MG, Nunes LR, Campaner M, Buck GA, Camargo EP (1999). Trypanosomatidae: *Phytomonas* detection in plants and phytophagous insects by PCR amplification of a genus-specific sequence of the spliced leader gene. Exp. Parasitol.

[18] Lodhi MA, Ye GN, Weeden NF, Reisch BI (1994). A simple and efficient method for DNA extraction from gravepine cultivars, *Vitis* species and *Ampelopsis*. Plant Mol. Biol. Rep.

[19] Zidani S, Ferchichi A, Chaieb M (2005). Genomic DNA extraction method from pearl millet (*Pennisetum glaucum*) Leaves. Afr. J. Biotechnol.

[20] Couch JA, Fritz PJ (1990). Isolation of DNA from plants high in polyphenolics. Plant Mol. Biol. Rep.

[21] Kim CS, Lee CH, Shin JS, Chung YS, Hyung NI (1997). A simple and rapid method for isolation of high quality genomic DNA from fruit trees and conifers using PVP. Nucleic Acids Res.

[22] Horne EC, Kumpatla SP, Patterson MG, Thompson SA (2004). Improved high-throughput sunflower and cotton genomic DNA extraction and PCR fidelity. Plant Mol. Biol. Rep.

[23] Friar EA (2005). Isolation of DNA from plants with large amounts of secondary metabolites. Method Enzymol.

[24] Padmalatha K, Prasad MNV (2006). Optimization of DNA isolation and PCR protocol for RAPD analysis of selected medicinal and aromatic plants of conservation concern from Peninsular India. Afr. J. Biotechnol.

[25] Arif IA, Bakir MA, Khan HA, Ahamed A, Al Farhan AH, Al Homaidan AA, Al Sadoon M, Bahkali AH, Shobrak M (2010). A simple method for DNA extraction from mature date palm leaves: impact of sand grinding and composition of lysis buffer. Int. J. Mol. Sci.

[26] Chen DH, Ronald PC (1999). A rapid DNA minipreparation method suitable for AFLP and other PCR applications. Plant Mol. Biol. Rep.

[27] Li JT, Yang J, Chen DC, Zhang XL, Tang ZS (2007). An optimized mini-preparation method to obtain high-quality genomic DNA from mature leaves of sunflower. Gen. Mol. Res.

[28] Feeley CJ, Hart ER, Thompson JR, Harrington TC (2001). Ocurrence, associated symptoms, and potential insect vectors of the ash yellows phytoplasma in Iowa, U.S. J. Arboric.

[29] Doyle JJ, Doyle JL (1990). Isolation of plant DNA from fresh tissue. Focus.

[30] Puchooa D, Venkatasamy K (2005). A protocol for the isolation of DNA from *Trochetia boutoniana*. Int. J. Agric. Biol.

[31] Michiels A, van den Ende W, Tucker M, van Riet L, van Laere A (2003). Extraction of high-quality genomic DNA from latex-containing plants. Anal. Biochem.

[32] Calderón CN (2010).

